# An innovative multi-modal retinal imaging system for *in vivo* retinal detection in small animals

**DOI:** 10.3389/fopht.2023.1251328

**Published:** 2023-09-28

**Authors:** Zhengyuan Tang, Tianze Zhao, Ji Ren, Kuan Zhang, Qi Yin, Teng Zhang, Hui Zhang, Tianyu Dong, Pengfei Zhang, Jie Zhang

**Affiliations:** ^1^ Advanced Ophthalmology Laboratory (AOL), Robotrak Technologies, Nanjing, Jiangsu, China; ^2^ Ophthalmology Department, Tri-Apex Laboratories Co., LTD, Nanjing, Jiangsu, China; ^3^ School of Optoelectronic Engineering and Instrumentation Science, Dalian University of Technology, Dalian, China

**Keywords:** reflectance retinal imaging, fluorescein angiography, optical coherence tomography, optical coherence tomography angiography, confocal scanning laser ophthalmoscopy, small animal retinal imaging

## Abstract

This paper presents an innovative retinal imaging system tailored for *in vivo* fundus detection in small animals. This system integrates Scanning Laser Ophthalmoscopy (SLO) and optical Coherence Tomography (OCT) techniques, enabling the simultaneous generation of images from various modalities, including SLO reflectance, SLO fluorescein angiogram, OCT, and OCT angiogram. The existing multi-modal retinal imaging systems generally encounter limitations such as the inability to detect peripheral lesion areas attributed to small Field of View (FOV) design and susceptibility to sample motion due to slow data acquisition speed. To address these challenges, it’s essential to underscore that this proposed system offers a range of notable advantages, including its compact design, the capacity for widefield imaging with a FOV of up to 100°, and a rapid OCT A-scan rate of 250 kHz, notably exceeding the capabilities of pre-existing multi-modal retinal imaging systems. Validation of the system involved imaging the eyes of normal wild-type mice and diseased mice afflicted with retinal detachment and choroidal neovascularization (CNV). The favorable imaging results demonstrate the system’s reliability in identifying retinal lesions in small animals.

## 1 Introduction

Animal retinal research plays a crucial role in advancing ophthalmology by providing valuable insights and facilitating the translation of research findings into clinical practice, ultimately benefiting patients with retinal disorders Junod et al. (1); Nakhooda et al. (2); Dobi et al. (3); Fletcher et al. (4). As technology advances, non-invasive imaging techniques have emerged as crucial tools for *in vivo* fundus research. Among these techniques, Scanning Laser Ophthalmoscopy (SLO) and Optical Coherence Tomography (OCT) stand out as two prominent methods.

SLO utilizes a raster scanning laser to sequentially illuminate elements on the retina, enabling pointby-point imaging of the retina Webb and Hughes (5); Zhang et al. (6); Fischer et al. (7). Two important SLO imaging modalities are reflectance retinal imaging and fluorescent angiography Seeliger et al. (8); Gramatikov (9). Reflectance retinal imaging is a conventional method to offer a direct capture of reflected light from the retina, which provides a macroscopic view of the retinal structures, such as the optic nerve head, blood vessels, and macula. On the other hand, FA involves the injection of a fluorescent dye into the bloodstream. The dye circulates through the blood vessels of the retina, allowing the visualization of retinal vasculature and the assessment of blood flow. FA provides imaging of smaller vessels such as capillaries that conventional reflectance retinal imaging cannot achieve.

OCT utilizes low-coherence light to create a three-dimensional reconstruction of the retina Aumann et al. (10); Fischer et al. (11). Our OCT channel comprises three imaging formats: OCT B-scan, OCT enface, and OCT angiogram. OCT enface generates a lateral two-dimensional projection image from the three-dimensional retinal volume, similar to the reflectance retinal imaging used in SLO. OCT B-scan offers a high-resolution cross-sectional view of retinal layers, facilitating the assessment of retinal thickness, morphological changes, and pathological features. OCT angiogram is generated using Optical Coherence Tomography Angiography (OCTA) technique, an extension derived from OCT De Carlo et al. (12). OCTA allows depth-resolved visualization of retinal vasculature without requiring the injection of a fluorescent contrast agent.

The integration of the aforementioned SLO and OCT imaging modalities provides researchers with comprehensive information about the structure, function, and blood flow of the retina in small animal models. This facilitates the study of various retinal diseases, including glaucoma, diabetic retinopathy, macular degeneration, retinopathy of prematurity, retinal vascular leakage, retinal detachment, and diabetic macular edema AbràCheck that all equations and special characters are displayed correctly.moff et al. (13); Eladawi et al. (14); Venkat and Sharma (15). Nowadays, retinal imaging systems that combine the SLO and OCT imaging modalities are not uncommon in research laboratories and commercial markets. Nonetheless, it’s important to note that there are relatively few retinal imaging systems tailored specifically for small animals in comparison to those designed for humans. Small animal eyes have smaller pupil sizes and significantly shorter axial lengths than human eyes. Consequently, the optical system’s imaging lenses must be purposefully designed to accommodate the imaging of small animal fundus. Additionally, some retinal imaging systems integrate different imaging modalities into separate modules, potentially resulting in larger and bulkier setups.

In this paper, we tackle these challenges by designing a compact module for a small animal retinal imaging system that combines SLO and OCT techniques to integrate all imaging modalities mentioned. Our designed system ensures that the images produced from all modalities share an identical wide FOV of up to 100°. Supported by an OCT A-scan rate of 250 kHz, the system rapidly generates images from all modalities (except OCT angiogram) and offers a real-time preview. Furthermore, since acquiring OCTA data relies on the repeated capture of multiple OCT A-scans or B-scans, the fast A-scan rate significantly enhances the efficiency of acquiring the bulk data required for OCTA, leading to improved quality of angiograms by reducing motion artifacts Sampson et al. (16). The optical structure of the proposed system that enables multi-modal imaging is detailed in Section 2. To validate the system’s performance, an experiment involving multi-modal imaging of the retinas of normal wild-type mice, as well as mice modeled with retinal detachment and CNV, has been designed. The experimental details and the resultant retinal images from all modalities are indicated in Section 3. Section 4 offers a brief examination of the advantages our proposed retinal imaging system offers in contrast to other multi-modal retinal imaging setups, and explicitly demonstrates the forthcoming endeavors aimed at enhancing this innovative retinal imaging system.

## 2 Structure of optical system

**Figure 1:**
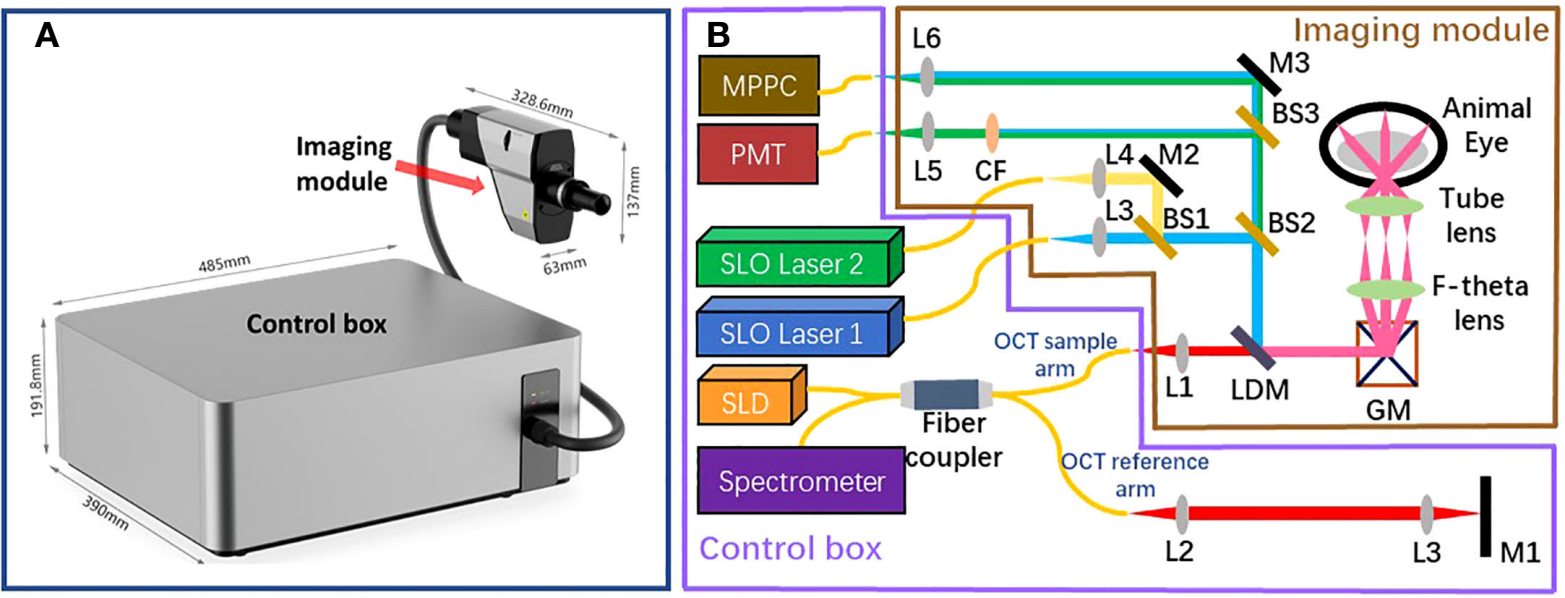
The structure of the proposed retinal imaging system that consists of a control box and an imaging module **(A)** The proposed retinal imaging system after assembling. **(B)** The system schematic of the proposed retinal imaging system: L, lens; M, mirror; GM, a pair of Galvo-mirrors; LDM, long-pass dichroic mirror; BS, beamsplitter; CF. color filter.

The assembled retinal imaging system and system schematic are depicted in **Figures 1A, B**, respectively. This system consists of two components: a control box and an imaging module. The control box accommodates light source, detectors, the OCT reference arm, and all associated electronic components. Optical fibers are utilized to couple this control box and the imaging module, which enables the transmission of excited light into the imaging module and facilitates the collection of back-scattered, back-reflectance, and epi-fluorescence signals from the retina by detectors. The imaging module is meticulously engineered to possess a compact form factor, measuring 328.6mm × 63mm × 137mm, and weighing only 0.8kg. This compact design enables easy attachment to a 5-axis manual stage for pupil alignment.

The imaging module comprises an OCT light source channel and two SLO light source channels. The OCT channel utilizes a broadband 850±30 nm superluminescent diode (SLD) as the light source, providing an axial resolution of 3.9 *µ*m (in tissue) for OCT B-scan imaging. The other two SLO channels are compatible with custom laser source options of different wavelengths, as long as the laser wavelength and the corresponding fluorescence emission wavelength of interest are shorter than SLD’s wavelength range. Thereby, the SLO channels are designed to accommodate various fluorescent contrast agents, such as Fluorescein Sodium Dye, Green Fluorescent Protein (GFP), Yellow Fluorescent Protein (YFP), and Red Fluorescent Protein (RFP). All beams from these three channels are collimated, aligned, and combined into a co-axis optical path before propagating towards a pair of galvo-mirrors. This configuration ensures that all SLO and OCT imaging modalities have the same FOV. Subsequent to the Galvo-mirrors, a bespoken pair of apochromatic F-theta scan lens and tube lens, designed in accordance with the small animal eye model suggested in Ref. Gardner et al. (17), directs the light to focus on the retina with a FOV of up to 100°.

All optical signals generated from the retina return to the imaging module, tracing the path they originated from, until the SLO beam is distinguished from the OCT beam using a long-pass dichroic mirror. The separated SLO beam is subsequently divided into two beam paths by using a beamsplitter (**BS2** in **Figure 1B**). In one beam path, the SLO beam is directed toward a Multi-Pixel Photon Counter (MPPC) detector to generate the retinal reflectance image. In another beam path, multiple color filters are attached in a filter wheels cage. Based on the used laser source and the fluorescent contrast agent, the appropriate color filter could be chosen to extract the emission fluorescence signal at the specific wavelength band. A Photomultiplier tube (PMT) detector is used to capture the fluorescence signal and generate the fluorescein angiogram. It is worth noting that fiber optic coupling is utilized for both the MPPC detector and PMT detector to collect signals. Thereby, the 25 *µ*m fiber core acts in a similar manner to a confocal aperture, effectively blocking out-of-focus light and enhancing imaging contrast.

**Figure 2:**
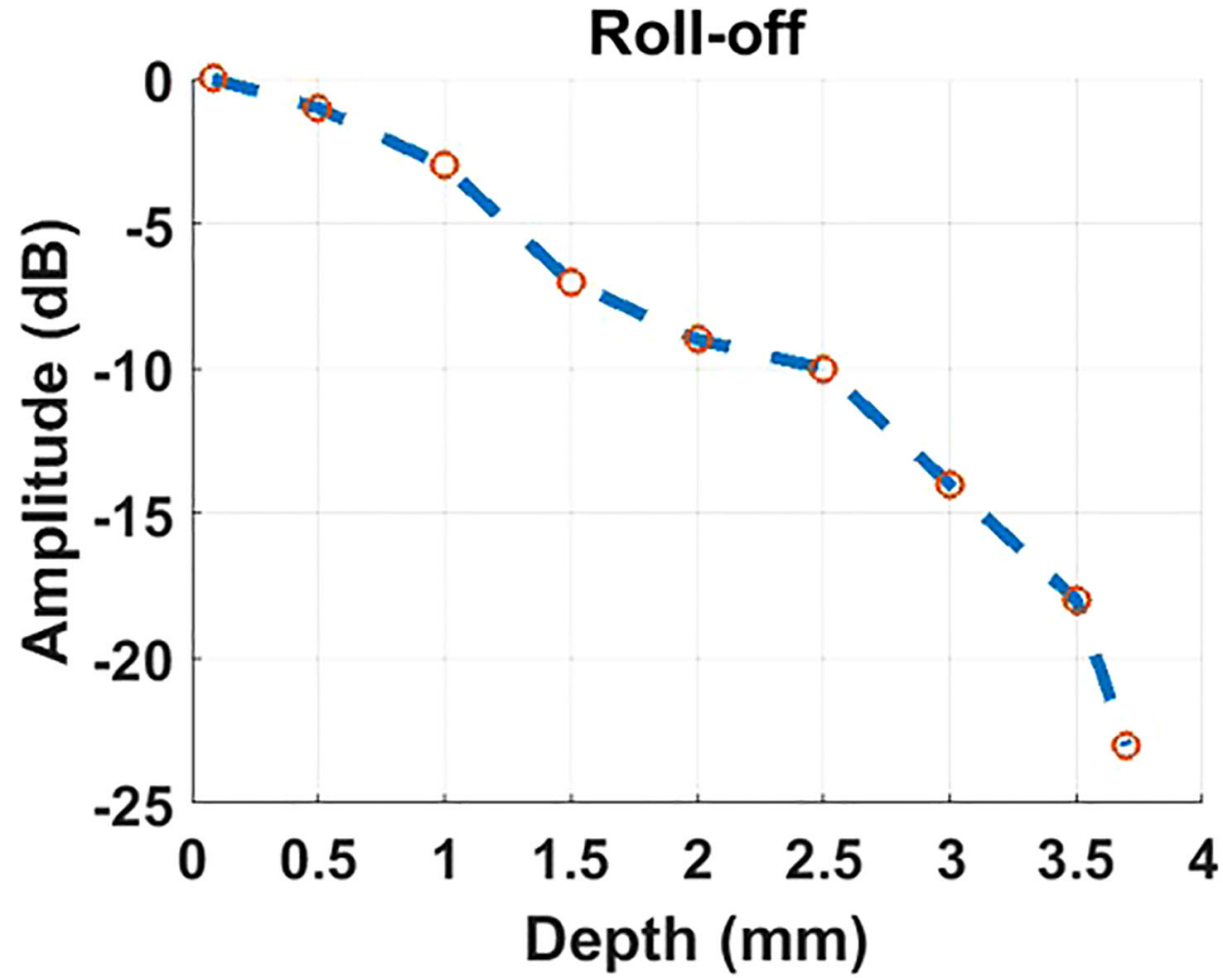
The profile of roll-off sensitivity versus imaging depth results from the OCT channel of our proposed imaging system.

The beam originating from the OCT sample arm retraces its original path through the control box and merges with the reference beam to create interference. The reference beam’s optical path length, adjustable through automatic motor control, enables the resulting interference signal to be depth-resolved. Subsequently, the interference light is directed into a spectrometer to generate a spectrum for further processing using Fourier domain-OCT (FD-OCT) techniques Choma et al. (18); De Boer et al. (19); Leitgeb et al. (20). The spectrometer employed in the proposed optical system has the capability to record a 2048-pixel spectrum within a wavelength range of 790 - 883nm, with a bandwidth of 93 nm. This allows for the generation of an A-scan comprising 1024 pixels, providing an imaging depth of 3.78 mm (in air). The camera integrated into the spectrometer permits an A-scan collection speed of up to 250 kHz, offering one of the fastest data acquisition speeds in the field of FD-OCT. Accordingly, Given the utilization of a lateral scan with dimensions of 512 x 512 pixels, this imaging system delivers a data collection time of 2.05 ms for a single OCT B-scan and 1.1 seconds for the acquisition of three-dimensional OCT C-scan data. The high A-scan rate is also particularly advantageous for OCTA, which necessitates the acquisition of a substantial amount of data through a recording of repetitive A-scans or B-scans De Carlo et al. (12); Kashani et al. (21). The rapid A-scan rate significantly reduces motion artifacts that can compromise the quality of OCTA images. Through the use of the specular reflection method Agrawal et al. (22), the sensitivity of the OCT channel was tested to reach up to 108 dB. The profile of sensitivity roll-off over the imaging depth is illustrated in **Figure 2**. Considering the requirement for the FD-OCT system to possess a sensitivity exceeding 100 dB, as indicated in Ref. Aumann et al. (10), the OCT channel within this system can be confirmed to deliver an image of satisfactory quality.

## 3 Experiment for mouse retinal imaging

### 3.1 Sample preparation

Two male C57Bl/6 mice, aged 7 weeks, were raised in the Tri-Apex Experimental Animal Center. The feeding conditions and dietary standards for these mice comply with the ARVO Guidelines for the Management and Use of Laboratory Animals. These two mice are respectively utilized to make the CNV model and the retina detachment model.

The CNV mouse model was made by laser photocoagulation to rupture the Bruch membrane. The mice were anesthetized and placed on the platform of the Slit lamp. Prior to the laser photocoagulation, the mice were anesthetized and positioned on a slit lamp platform. Tropicamide was used to dilate the pupils. A contact ophthalmoscope was then positioned in front of the mouse’s cornea to deliver 532 nm laser light to the fundus. Laser photocoagulation was performed by applying three laser points around the optic nerve of the left eye of each mouse. Successful laser photocoagulation was determined by the observation of small air bubbles in front of the laser spot. To maintain the well-being of the mouse eyes, antibiotic eye ointment was applied to keep them moist.

The Retina detachment mouse model was made using the following steps. First, a 30G injection needle was used to puncture the wall of the eyeball, approximately 0.5 mm from the corneoscleral limbus. To maintain the moisture of the ocular surface, eye ointment was applied. Next, a glass slide coated with eye ointment was placed on the surface of the eyeball, and the fundus was observed under a microscope. A micro-injector was inserted through the puncture site to reach the vitreous cavity. The needle of the micro-injector was gently pushed into the retina, and a drug solution was slowly injected until a bulge was observed in a specific area of the retina. The injection was then paused for approximately 10 seconds before slowly withdrawing the needle. Finally, a sterile cotton swab was lightly pressed against the puncture site for about 10 seconds to ensure proper sealing.

Additionally, a female wild-type C57Bl/6 mouse and a female wild-type ICR/CD1 mouse, both aged 8 weeks, purchased from Gempharmatech Biotech company, were also investigated in this experiment.

### 3.2 Pre-imaging preparation

Prior to conduct the retinal imaging experiment on mice, each mouse was anesthetized using 2% isoflurane in O_2_. Once the mouse’s heart rate slowed down to 0.5-1 beat/second, we applied a 50 *µ*L injection of 10% sodium fluorescein into the mouse’s peritoneum. The mouse was then carefully placed on a custom xyz translational platform and immobilized using a ’bite bar’ connected to a tube for continuous isoflurane supply. 1% tropicamide was used to dilate mouse’s pupil. After 6-minute interval, a saline solution was dropped onto the cornea to maintain moisture, and the mouse was then positioned in front of the imaging module of our proposed retinal imaging system. We then activated the blue laser and NIR light source. At the cornea position, we measured the power of the blue laser to be 200 *µ*W, and the power of the NIR source to be 2 mW. These power levels comply with the ocular laser safety limit specified by the American National Standard for Safe Use of Lasers (ANSI). Finally, we guided the 488nm blue laser and NIR light into the mouse’s pupil. Owing to the co-axis design of incident light paths, Both SLO and OCT images possess an identical field of view (FOV) and can be synchronized for scanning. All images were acquired through 100° widefield scans, featuring a lateral plane resolution of 512 × 512 pixels. To minimize the influence of breathing and reduce noise, we performed 5 repetitions of SLO and OCT captures and then computed the average of these repetitions during the subsequent numerical pre-processing. The duration of SLO and OCT data collection is 5.5 seconds. Regarding the reconstruction of OCTA, we conducted four repetitions of B-scan recordings and three repetitions of OCT volumetric captures, all completed within a data acquisition period of 13.2 seconds. The reconstructed OCT angiograms were generated through the utilization of the optical microangiography (OMAG) algorithm, as detailed in previous works by Wang et al. (23); Wang (24).

### 3.3 Retinal imaging for wild-type mice

**Figure 3:**
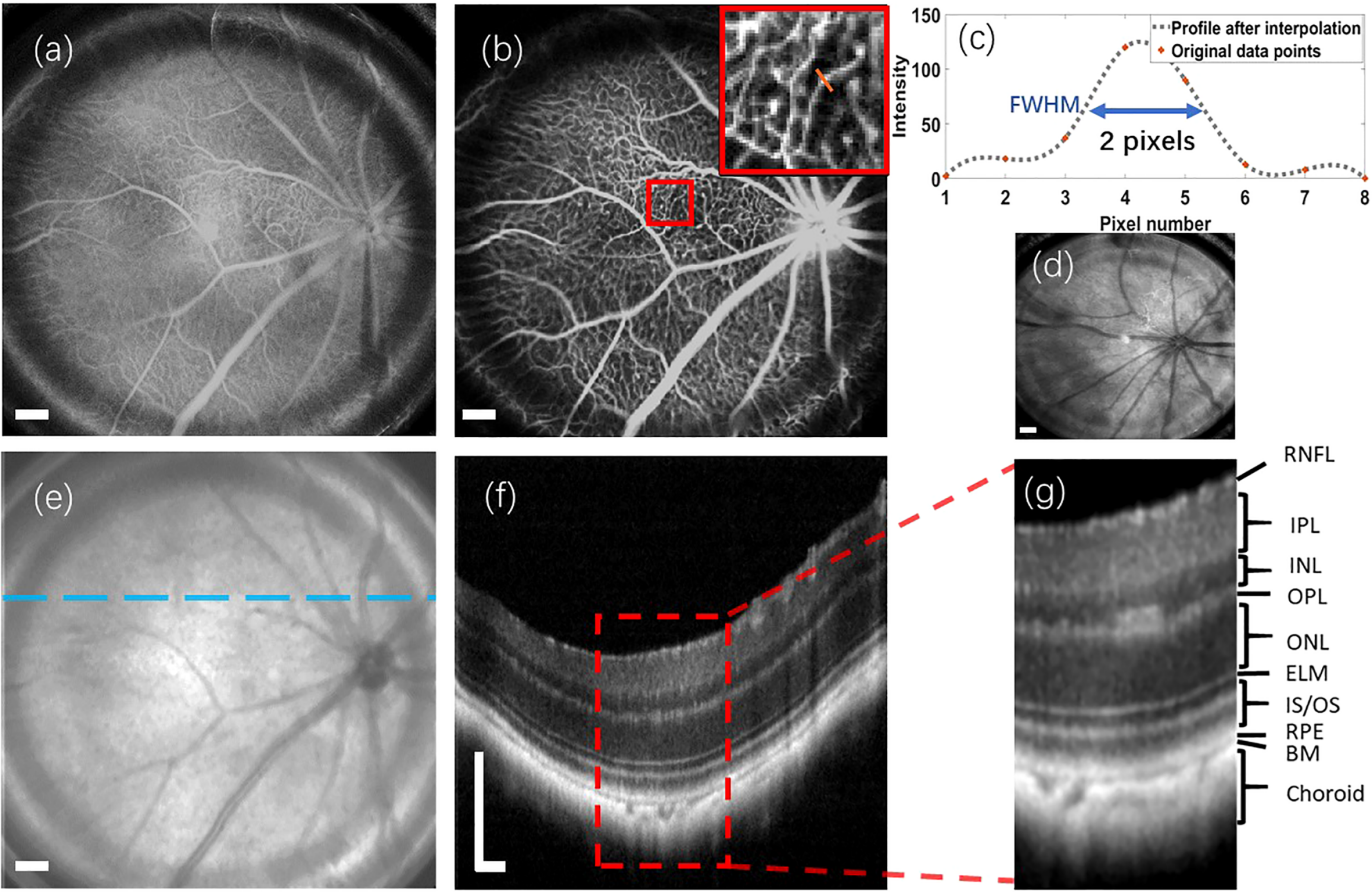
Multi-modal retinal images captured from a wild-type C57Bl/6 mouse, as well as a retinal reflectance image of a wild-type ICR/CD1 mouse. **(A)** Retinal reflectance image of the C57Bl/6 mouse. **(B)** Fluorescein angiogram of the C57Bl/6 mouse. **(C)** The interpolation profile along the orange line in the inset of **(B)**. **(D)** Retinal reflectance image of the ICR/CD1 mouse without injecting the sodium fluorescein. **(E)** An OCT enface of C57Bl/6 mouse. **(F)** OCT B-scan of C57Bl/6 mouse, which corresponds to the lateral location marked by the blue line in **(E)**. **(G)** An enlarged view of the region enclosed by a red rectangle in **(F)**. Scale Bar, 200 *µ*m.

To assess the performance of the proposed retinal imaging system and exclude any potential disease-related effects on image quality, we initially utilized the system to capture a group of multi-modal retinal images from the wild-type C57Bl/6 mouse. **Figures 3A, B** illustrate the retinal reflectance image and fluorescein angiogram, respectively, of the wild-type C57Bl/6 mouse. Resulting from the flow of sodium fluorescein in the retinal blood vessels, the fluorescein angiogram offers a visualization of different sizes of vessel branches, including central retinal artery and vein, superior branches, inferior branches, distal branches, and capillaries. Due to the presence of the crystalline lens and retina within the eye, accurately measuring the lateral resolution for *in vivo* retinal imaging is challenging. However, the visualization of these small capillaries provides a means to estimate the system’s lateral resolution.

In **Figure 3B**, we draw attention to an inset in the top-right corner, highlighting the magnified region surrounded by the red square in the fluorescein angiogram. A spline interpolated intensity profile along the orange line traversing a capillary is depicted in **Figure 3C**. Measuring the Full width of half maximum (FWHM) of this interpolated intensity profile yields a value of 2 pixels (∼12.6 *µ*m). This FWHM of the interpolated intensity profile, as per the knowledge of Fourier Optics Goodman (25), emerges as a result of convolving the marked capillary size with the FWHM of the point spread function (PSF) of the proposed optical system. Simultaneously, the optical system’s lateral resolution conventionally corresponds to the FWHM of its PSF. According to the findings in Ref. Joseph et al. (26), where retinal capillaries’ diameter ranges between 3-7 *µ*m, indicative of the system’s lateral resolution can be reasonably inferred to be under 10 *µ*m.

It’s important to note that the retinal reflectance channel also captures a strong fluorescent signal. As depicted in **Figure 3A**, the bright vessels seen in the retinal reflectance image result from the combination of both back-reflected and fluorescent signals. To demonstrate the capabilities of the Retinal reflectance imaging utilized by the proposed system, a retinal reflectance retinal image of a wild-type ICR/CD1 mouse, without injecting the sodium fluorescein, is showcased in **Figure 3D**. In this retinal reflectance image, vessels manifest as dark features, while some thin superior branches and smaller vessels remain invisible due to the weak nature of their reflectance signals.

The OCT enface image is displayed in **Figure 3E**. OCT enface image is generated from superimposing the 2D lateral planes in reconstructed 3D tomographic volume, which has a similar imaging effect to the retinal reflectance image. Along the transverse blue line marked in **Figure 3E**, an averaged OCT B-scan with visualization of different retinal layers is depicted in **Figure 3F**. This OCT B-scan provides a visualization of different retinal layers. The abbreviations for each retinal layer mentioned in the figures and content of this paper are listed and explained in **Table 1**. After enlarging the red rectangular area in **Figure 3G**, it can be observed in **Figure 3G** that nine retinal layers and the choroid can be distinguished, which meets the requirements for retinal cross-section observation in the field of ophthalmic pathology.

**Table 1:** The abbreviations of mouse retina layers.

RNFL	Retinal nerve fiber layer
IPL	Inner plexiform layer
INL	Inner nuclear layer
OPL	Outer plexiform layer
ONL	Outer nuclear layer
ELM	External limiting membrane
IS/OS	Inner/outer segment
RPE	Retinal pigment epithelium
BM	Basement membrane

**Figure 4:**
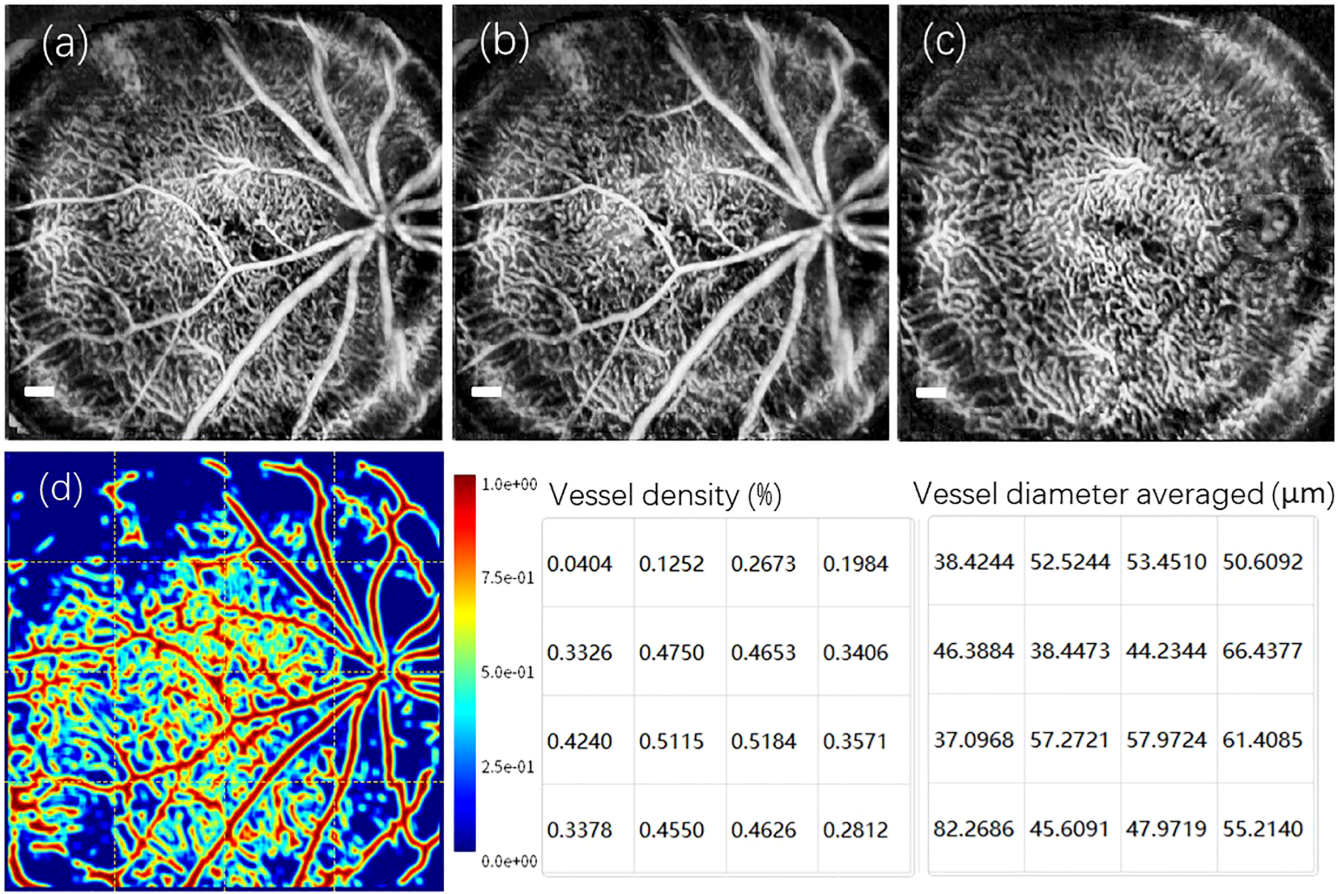
A set of reconstructed enface OCTA images captured from the retina of wild-type C57Bl/6 mouse. **(A)** The enface maximum intensity projection of the angiogram from the inner retina between the RNFL and the ONL. **(B)** The enface vascular projection from the retinal layers: RNFL and IPL. **(C)** The enface vascular projection from the Retinal layers: INL and OPL. **(D)** The heat map corresponding to the classification of vessel branches, accompanied by a vessel density chart and an averaged vessel diameter chart derived from the grids in heat map. Scale Bar, 200 *µ*m.

Derived from the same wild-type C57Bl/6 mouse, a set of enface OCTA retinal angiograms is presented in **Figure 4**. Notably, these OCTA enface images share the same FOV as the enface image depicted in **Figure 3**. **Figure 4A** depicts the maximum intensity projection of the angiogram data from the inner retina, specifically between the retinal nerve fiber layer (RNFL) and the outer nuclear layer (ONL). By utilizing the reconstructed 3D volumetric OCTA retinal data, a numerical segmentation algorithm can be utilized to obtain vascular information from a desired single retinal layer or multiple layers. **Figure 4B** shows the enface projection of the angiogram from the retinal layers between RNFL and Inner plexiform layer (IPL). Furthermore, **Figure 4C** displays the enface projection of the angiogram from the Inner nuclear layer (INL) and Outer plexiform layer (OPL). In **Figure 4D**, a heat map illustrating the categorization of vessel branches is depicted. This heat map utilizes distinct colors to differentiate and label the central retinal artery and vein, superior branches, inferior branches, and other smaller branches. From the heat map’s grid layout, vessel density and the average vessel diameter for each grid are computed quantitatively and subsequently visualized in the two charts shown in **Figure 4**. It is worth noting that motion artifacts caused by the mouse’s breathing and signal deterioration in the peripheral FOV due to defocus are two challenges that currently limit the quality of OCT angiograms. Addressing these issues requires the development of a more robust breath-removal registration algorithm and a more accurate mouse eye model.

### 3.4 Retinal imaging for disease-model mice

**Figure 5:**
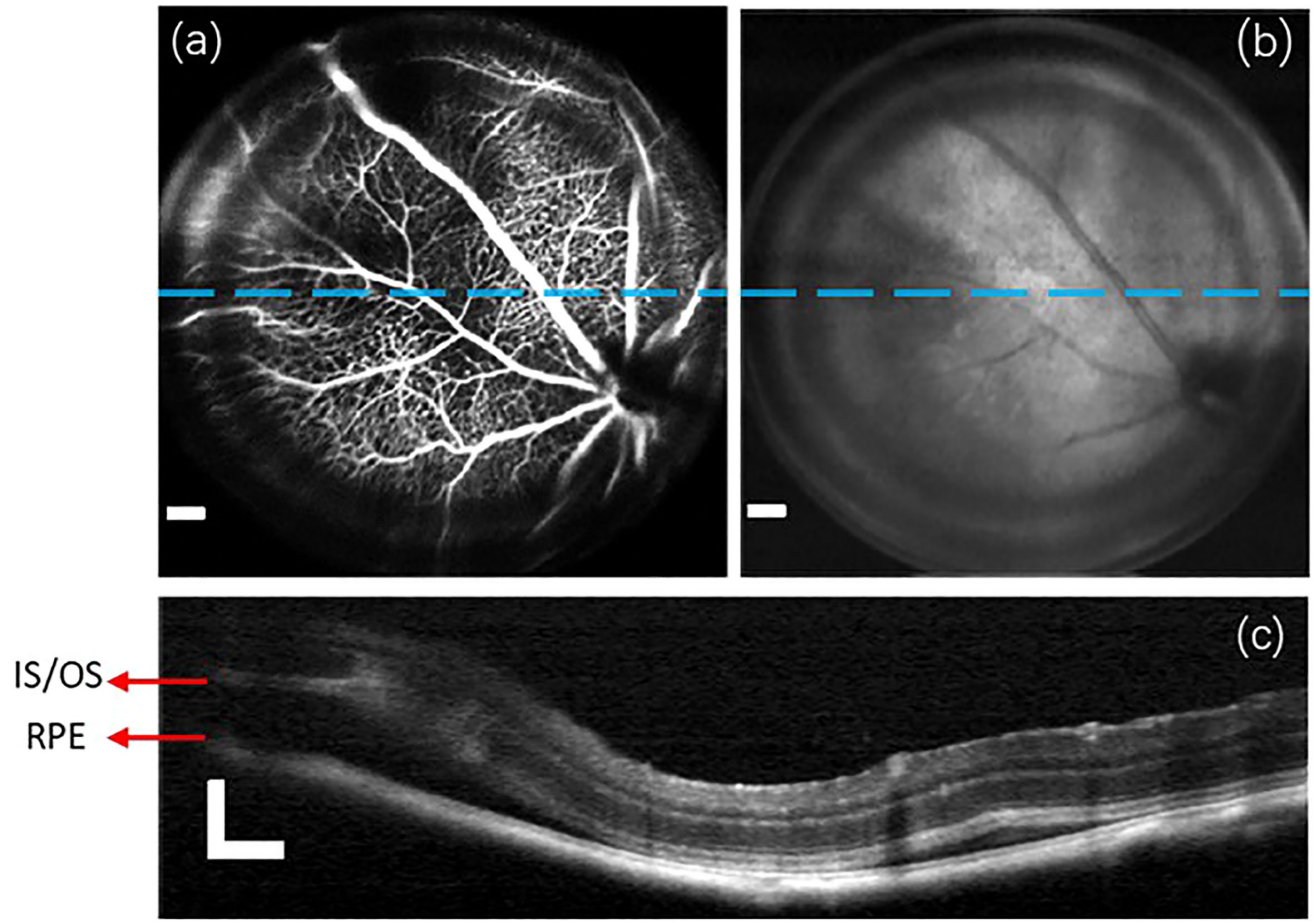
A group of retinal images captured from the C57Bl/6 mouse of retinal detachment model. **(A)** Fluorescein angiogram. **(B)** OCT enface image. **(C)** the OCT B-scan images obtained from the lateral location indicated by blue lines in **(A, B)**. Scale Bar, 200*µ*m.

In this section, we highlight the capabilities of the proposed imaging system in observing and diagnosing retinal diseases. Initially, we performed multi-modal imaging on the C57Bl/6 mouse of retinal detachment model. The resulting group of retinal images is presented in **Figure 5**. Due to the defocused imaging caused by retinal detachment, the signal from the detachment region is reduced. Consequently, the detachment region appears dark in both the fluorescein angiogram (**Figure 5A**) and OCT enface image (**Figure 5B**). OCT B-scan imaging provides a detailed axial targeting on the detachment region, as evidenced in **Figure 5C** by the clear separation between RPE and IS/OS layers.

**Figure 6:**
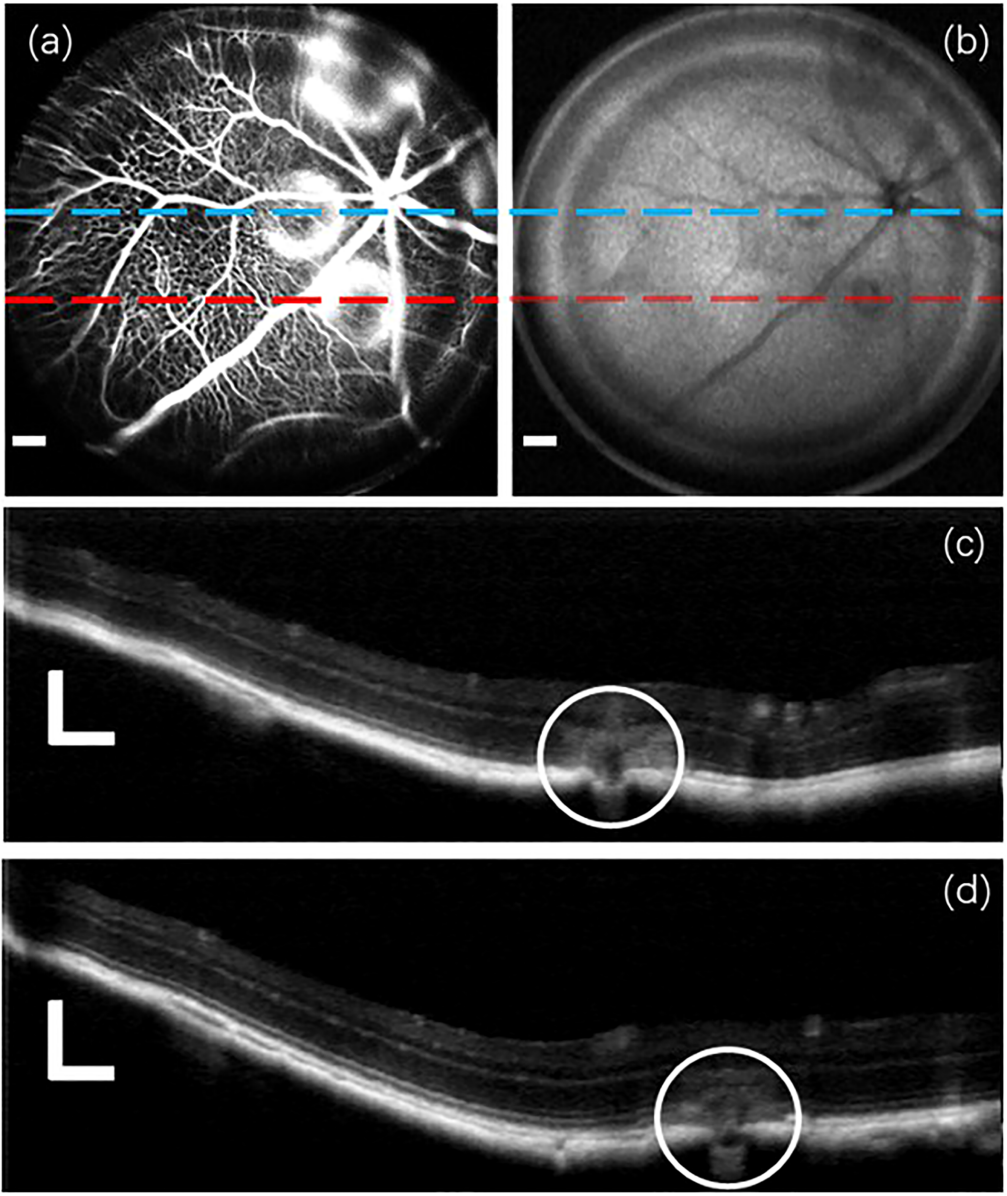
A group of retinal images captured from the C57Bl/6 mouse of CNV model. **(A)** Fluorescein angiogram. **(B)** OCT enface image. **(C, D)** OCT B-scan images acquired from two distinct lateral positions marked by blue and red lines, respectively, in the previous **(A, B)**. Scale Bar, 200*µ*m.

Next, a group of retinal images from the C57Bl/6 mouse of CNV model are demonstrated in **Figure 6**. The fluorescein angiogram in **Figure 6A** easily identifies the lateral regions of fluorescein leakage, depicted as bright circular patterns. However, the leakage regions in OCT enface image in **Figure 6B** is dark, because RPE and choroid of strong backscattering properties are damaged by photocoagulation. The two OCT B-scan images acquired from two designated lines on the lateral plane are exhibited in **Figures 6C, D**, respectively. The leakage regions detected in the B-scan images are demarcated by white circles.

## 4 Discussion

In this article, an *in vivo* multi-modal retinal imaging system that combines SLO and OCT techniques designed for small animals is proposed. Utilizing this system, a variety of multi-modal images, including SLO reflectance image, SLO fluorescein angiogram, OCT enface, OCT B-scan, and/or OCT angiogram, were reconstructed from both normal wild-type mice and diseased mice of retinal detachment and choroidal neovascularization (CNV). These reconstructed images serve as validation, confirming the effectiveness of the proposed system.

It is important to recognize that this proposed system is not the earliest retinal imaging system designed for small animals to integrate the aforementioned imaging modalities. Earlier works by Issaei et. al. Issaei et al. (27), Liu et al. Liu et al. (28) and Zhang et al. Zhang et al. (6) also involved the development of multi-modal retinal imaging setups that combined SLO and OCT to explore small animal fundus. However, it is noteworthy that the field of view (FOV) and A-scan collection rate in their systems were all below 60° and 50 kHz, respectively. This distinction allows our proposed retinal imaging system to exhibit markedly enhanced performance, enabling the acquisition of multi-modal images with broader FOV in a significantly shorter duration. In comparison to existing commercial multi-modal retinal imaging setups that contain the above imaging modalities, such as the HRA+OCT Spectralis system by Heidelberg Engineering*^TM^* and the Micron System by Phoenix-Micron*^TM^*, our proposed system also demonstrates its superiority. Notably, the Micron System utilizes the Fundus camera technique to produce the retinal reflectance image and fluorescein angiogram, rather than employing SLO. Nevertheless, the optical principles underlying the generation of retinal reflectance and fluorescein angiogram remain consistent. The HRA+OCT Spectralis system offers an A-scan collection rate of 85 kHz and an imaging FOV of 55°, while the Micron system possesses an A-scan collection rate of 18 kHz and an imaging FOV of 50°. Furthermore, the Micron system utilizes a cumbersome design where various imaging modalities are incorporated into distinct modules. In comparison to these two established commercial multi-modal systems, it’s necessary to reiterate that the retinal imaging system introduced in this paper features a compact design, an OCT A-scan rate of 250 kHz, an imaging FOV of 100°. This enables a space-efficient arrangement and significantly reduces the time required for capturing images with a broader FOV.

Although the proposed system has yielded promising results in generating multi-modal images, there is still potential for enhancement in terms of image quality. The first area of focus is the removal of dark ring artifacts near the edge of FOV. These artifacts are present in SLO and OCT enface images, as well as the corresponding lateral positions in cross-sectional OCT B-scans. The cause of these artifacts has been identified as a fabrication error in the tube lens from the lens manufacturer. Replacing the tube lens with the correct one can solve this artifact problem.

The second area required for improvement is enhancing the image quality in the peripheral region of the wide FOV, which is a more challenging task. Due to variations in mouse types and ages, the curvature of the retina differs among individuals, making it less accurate to fit the mouse eye model used. This mismatch between the mouse eye model and the actual mouse eye can introduce aberrations and defocus in the peripheral region, resulting in an imaging deterioration and a signal collection reduction in that region. This mismatch particularly affects the imaging of the retinal external limiting membrane (ELM) due to its thin thickness and weak scattering properties. To address this issue, a large number of retinal images from different types and ages of mice will be recorded to create a database. The mouse eye model will be continuously optimized, and a pair of tube lens and scan lens will be designed to be compatible with the evolving mouse eye model.

Additionally, since the imaging module can integrate multiple laser inputs from lasers of different wavelengths, there is ongoing work to create pseudo-color SLO retinal reflectance images. Furthermore, it is worth noting that this retinal imaging system is not limited to mouse retina but can also be used for rat, guinea pig, and zebrafish retinas. Further research is underway to design specialized pairs of tube lens and scanning lens for retinal imaging in dogs, cats, and monkeys.

## Data availability statement

The original contributions presented in the study are included in the article/supplementary material. Further inquiries can be directed to the corresponding authors.

## Ethics statement

The animal study was approved by Institutional Animal Care and Use Committee. The study was conducted in accordance with the local legislation and institutional requirements.

## Author contributions

ZT takes responsibility for constructing optical setup and postprocessing the collected data. TZZ and JR align the optical path of this imaging system. HZ and TD raised the mice in their animal lab, and provide the experimental guidance. JZ made an overall plan for this experimental research. KZ designed the bespoken F-theta lens and tube lens in the imaging module. QY was responsible for coding the numerical processing algorithm. TZ designed the electronic system that connects the motors, computers and all optoelectronic components used in the system. PZ provided valuable instructions on OCT and SLO techniques, and help in developing the first generation of the prototype, and also help in how to do the mouse retina imaging and image processing. All authors contributed to the article and approved the submitted version.
